# Disease Evolution and Response to Rapamycin in Activated Phosphoinositide 3-Kinase δ Syndrome: The European Society for Immunodeficiencies-Activated Phosphoinositide 3-Kinase δ Syndrome Registry

**DOI:** 10.3389/fimmu.2018.00543

**Published:** 2018-03-16

**Authors:** Maria Elena Maccari, Hassan Abolhassani, Asghar Aghamohammadi, Alessandro Aiuti, Olga Aleinikova, Catherine Bangs, Safa Baris, Federica Barzaghi, Helen Baxendale, Matthew Buckland, Siobhan O. Burns, Caterina Cancrini, Andrew Cant, Pascal Cathébras, Marina Cavazzana, Anita Chandra, Francesca Conti, Tanya Coulter, Lisa A. Devlin, J. David M. Edgar, Saul Faust, Alain Fischer, Marina Garcia-Prat, Lennart Hammarström, Maximilian Heeg, Stephen Jolles, Elif Karakoc-Aydiner, Gerhard Kindle, Ayca Kiykim, Dinakantha Kumararatne, Bodo Grimbacher, Hilary Longhurst, Nizar Mahlaoui, Tomas Milota, Fernando Moreira, Despina Moshous, Anna Mukhina, Olaf Neth, Benedicte Neven, Alexandra Nieters, Peter Olbrich, Ahmet Ozen, Jana Pachlopnik Schmid, Capucine Picard, Seraina Prader, William Rae, Janine Reichenbach, Stephan Rusch, Sinisa Savic, Alessia Scarselli, Raphael Scheible, Anna Sediva, Svetlana O. Sharapova, Anna Shcherbina, Mary Slatter, Pere Soler-Palacin, Aurelie Stanislas, Felipe Suarez, Francesca Tucci, Annette Uhlmann, Joris van Montfrans, Klaus Warnatz, Anthony Peter Williams, Phil Wood, Sven Kracker, Alison Mary Condliffe, Stephan Ehl

**Affiliations:** ^1^Center for Chronic Immunodeficiency, Medical Center – University of Freiburg, Freiburg, Germany; ^2^Department of Pediatrics and Adolescent Medicine, Medical Center – University of Freiburg, Freiburg, Germany; ^3^Division of Clinical Immunology, Department of Laboratory Medicine, Karolinska Institute at Karolinska University Hospital Huddinge, Stockholm, Sweden; ^4^Research Center for Immunodeficiencies, Pediatric Center of Excellence, Children’s Medical Center, Tehran University of Medical Sciences, Tehran, Iran; ^5^San Raffaele Telethon Institute for Gene Therapy (SR-TIGET), Pediatric Immunohematology and Bone Marrow Transplantation Unit, IRCCS San Raffaele Scientific Institute, Milan, Italy; ^6^Research Department, Belarusian Research Center for Pediatric Oncology, Hematology and Immunology, Minsk, Belarus; ^7^Central Manchester University Hospitals NHS Foundation Trust, Manchester, United Kingdom; ^8^Division of Pediatric Allergy/Immunology, Marmara University, Istanbul, Turkey; ^9^Cambridge Centre for Lung Defense, Papworth Hospital, Cambridge, United Kingdom; ^10^Institute of Immunity and Transplantation, Royal Free Hospital, London, United Kingdom; ^11^University Department of Pediatrics, Bambino Gesù Children’s Hospital IRCCS, Rome, Italy; ^12^Department of Systems Medicine, University of Rome Tor Vergata, Rome, Italy; ^13^Department of Paediatric Immunology, Newcastle upon Tyne Hospital NHS Foundation Trust, Newcastle upon Tyne, United Kingdom; ^14^Internal Medicine, University Hospital of Saint-Etienne, Saint-Etienne, France; ^15^Biotherapy Department, Assistance Publique-Hôpitaux de Paris (AP-HP), Necker Children’s Hospital, Paris, France; ^16^Laboratory of Human Lymphohematopoiesis, INSERM UMR 1163, Imagine Institute, Paris, France; ^17^Paris Descartes-Sorbonne Paris Cité University, Paris, France; ^18^Department of Clinical Immunology, Addenbrookes Hospital, Cambridge, United Kingdom; ^19^Department of Medicine, University of Cambridge, Cambridge, United Kingdom; ^20^Regional Immunology Service, The Royal Hospitals & Queen’s University, Belfast, United Kingdom; ^21^NIHR Clinical Research Facility, University Hospital Southampton NHSFT, Southampton, United Kingdom; ^22^Department of Pediatric Immunology, Hematology and Rheumatology, Assistance Publique-Hôpitaux de Paris (AP-HP), Necker Children’s Hospital, Paris, France; ^23^INSERM UMR 1163, Imagine Institute, Paris, France; ^24^Pediatric Infectious Diseases and Immunodeficiencies Unit, Hospital Universitari Vall d’Hebron, Vall d’Hebron Research Institute (VHIR), Barcelona, Spain; ^25^Immunodeficiency Centre for Wales, University Hospital of Wales, Cardiff, United Kingdom; ^26^French National Reference Center for Primary Immune Deficiencies (CEREDIH), Necker Enfants Malades University Hospital, Assistance Publique-Hôpitaux de Paris, Paris, France; ^27^Department of Immunology, 2nd Faculty of Medicine Charles University and Motol University Hospital, Prague, Czechia; ^28^Department of Immunology, Research and Clinical Center for Pediatric Hematology, Oncology and Immunology, Moscow, Russia; ^29^Sección de Infectologıa, Rheumatología and Inmunodeficiencias, Unidad de Pediatria, Hospital Virgen del Rocıo, Instituto de Biomedicina de Sevilla (IBiS), Sevilla, Spain; ^30^Laboratory of Immunogenetics of Pediatric Autoimmunity, INSERM UMR 1163, Imagine Institute, Paris, France; ^31^Division of Immunology, University Children’s Hospital Zurich and Children’s Research Centre, University Zurich, Zurich, Switzerland; ^32^Study Center for Primary Immunodeficiencies, Necker-Enfants Malades Hospital, Assistance Publique-Hôpitaux de Paris (AP-HP), Necker Medical School, Paris, France; ^33^Laboratory of Lymphocyte Activation and Susceptibility to EBV Infection, INSERM UMR 1163, Imagine Institute, Paris, France; ^34^Wilhelmina Children’s Hospital, Utrecht, Netherlands; ^35^Department of Clinical Immunology and Allergy, St James’s University Hospital, Leeds, United Kingdom; ^36^Department of Infection, Immunity and Cardiovascular Science, University of Sheffield, Sheffield, United Kingdom

**Keywords:** activated phosphoinositide 3-kinase δ syndrome, PIK3CD, PIK3R1, registry, natural history, rapamycin

## Abstract

Activated phosphoinositide 3-kinase (PI3K) δ Syndrome (APDS), caused by autosomal dominant mutations in *PIK3CD* (APDS1) or *PIK3R1* (APDS2), is a heterogeneous primary immunodeficiency. While initial cohort-descriptions summarized the spectrum of clinical and immunological manifestations, questions about long-term disease evolution and response to therapy remain. The prospective European Society for Immunodeficiencies (ESID)-APDS registry aims to characterize the disease course, identify outcome predictors, and evaluate treatment responses. So far, 77 patients have been recruited (51 APDS1, 26 APDS2). Analysis of disease evolution in the first 68 patients pinpoints the early occurrence of recurrent respiratory infections followed by chronic lymphoproliferation, gastrointestinal manifestations, and cytopenias. Although most manifestations occur by age 15, adult-onset and asymptomatic courses were documented. Bronchiectasis was observed in 24/40 APDS1 patients who received a CT-scan compared with 4/15 APDS2 patients. By age 20, half of the patients had received at least one immunosuppressant, but 2–3 lines of immunosuppressive therapy were not unusual before age 10. Response to rapamycin was rated by physician visual analog scale as good in 10, moderate in 9, and poor in 7. Lymphoproliferation showed the best response (8 complete, 11 partial, 6 no remission), while bowel inflammation (3 complete, 3 partial, 9 no remission) and cytopenia (3 complete, 2 partial, 9 no remission) responded less well. Hence, non-lymphoproliferative manifestations should be a key target for novel therapies. This report from the ESID-APDS registry provides comprehensive baseline documentation for a growing cohort that will be followed prospectively to establish prognostic factors and identify patients for treatment studies.

## Introduction

Heterozygous gain-of-phosphoinositide 3-kinase (PI3K) δ-function mutations in *PIK3CD* or *PIK3R1* cause an autosomal-dominant primary immunodeficiency (PID) called activated phosphoinositide 3-kinase δ syndrome (APDS) or PASLI (p110-delta-activating mutation causing senescent T cells, lymphadenopathy, and immunodeficiency) 1 and 2, respectively (1–4). The main clinical and immunological characteristics of APDS 1 and 2 have been recently described in two major retrospective cohort studies (5, 6). Recurrent respiratory infections and benign lymphoproliferation emerged as key clinical aspects of the disease in both cohorts. Bronchiectasis was noted as a frequent complication with 60% in the APDS1 cohort and less frequently (18%) in the APDS2 cohort study. Additional immune dysregulation including cytopenias, glomerulonephritis, arthritis, and colitis was reported in these studies. An increased risk for lymphoma was also highlighted with 13% among the APDS1 patients and 28% in the APDS2 cohort. Non-immunological characteristics included neurodevelopmental delay (19% of APDS1 and 31% of APDS2) and growth impairment, especially among APDS2 patients (45%). Immunologically, hypogammaglobulinemia with increased IgM levels was frequent. B-cell lymphopenia, worsening with age, and expansion of transitional B cells were the main B-cell alterations. A reduction in the frequency of naïve CD4^+^ and CD8^+^ T cells with an increased frequency of effector/effector memory CD8^+^ T cells was reported. These first two important retrospective analyses of the disease illustrated clinical and immunological characteristics but did not address the dynamics of the disease evolution over time. Furthermore, although both reports showed that the majority of APDS patients receive supportive therapies in terms of immunoglobulin-replacement treatment (IGRT) or antimicrobial prophylaxes, data regarding immunosuppressive treatments were only reported for a limited number of patients. Here, we use an initial report from the European Society for Immunodeficiencies (ESID)-APDS prospective registry to address some of these questions.

## Methods

### The ESID-APDS Registry: Goals and Design

The ESID is a not-for-profit association whose aim is to improve knowledge in the field of PIDs (www.esid.org). The ESID Registry is an international Internet-based database for basic epidemiological (level 1), and more extensive disease-specific (level 3) data on patients with PID. The APDS Registry is the first prospective level 3 project that was initiated to better define the natural history of patients with APDS. The study is carried out in accordance with the recommendations of Section 15 of the Code of Conduct of the General Medical Council of Baden-Württemberg, Germany. The protocol was approved by the Ethics committee of the University of Freiburg (IRB approval No. ESID registry: 493/14; IRB approval No. APDS registry: 458/15). All subjects gave written informed consent in accordance with the Declaration of Helsinki. The goals of the project are to characterize disease evolution over time, to establish prognostic factors and biomarkers, to assess the impact of various treatment strategies, and to identify patients who could be eligible for novel treatments and interventions. Entry into the database requires an initial retrospective documentation, followed by yearly prospective follow-ups. Because of required patient consent, deceased patients cannot be registered. Each patient is evaluated at entry for eligibility by one of the three chief investigators to ensure that only patients with functionally validated APDS-associated mutations are registered. The APDS registry is supported by the pharmaceutical companies Novartis, GlaxoSmithKline, and UCB UK, who financed development and maintenance of the online level 3-documentation-section for APDS as well as project management including ethics submission in all participating countries, data management, and quality controls.

## Results

### Disease Manifestations and Their Evolution Over Time

**Figure 1:**
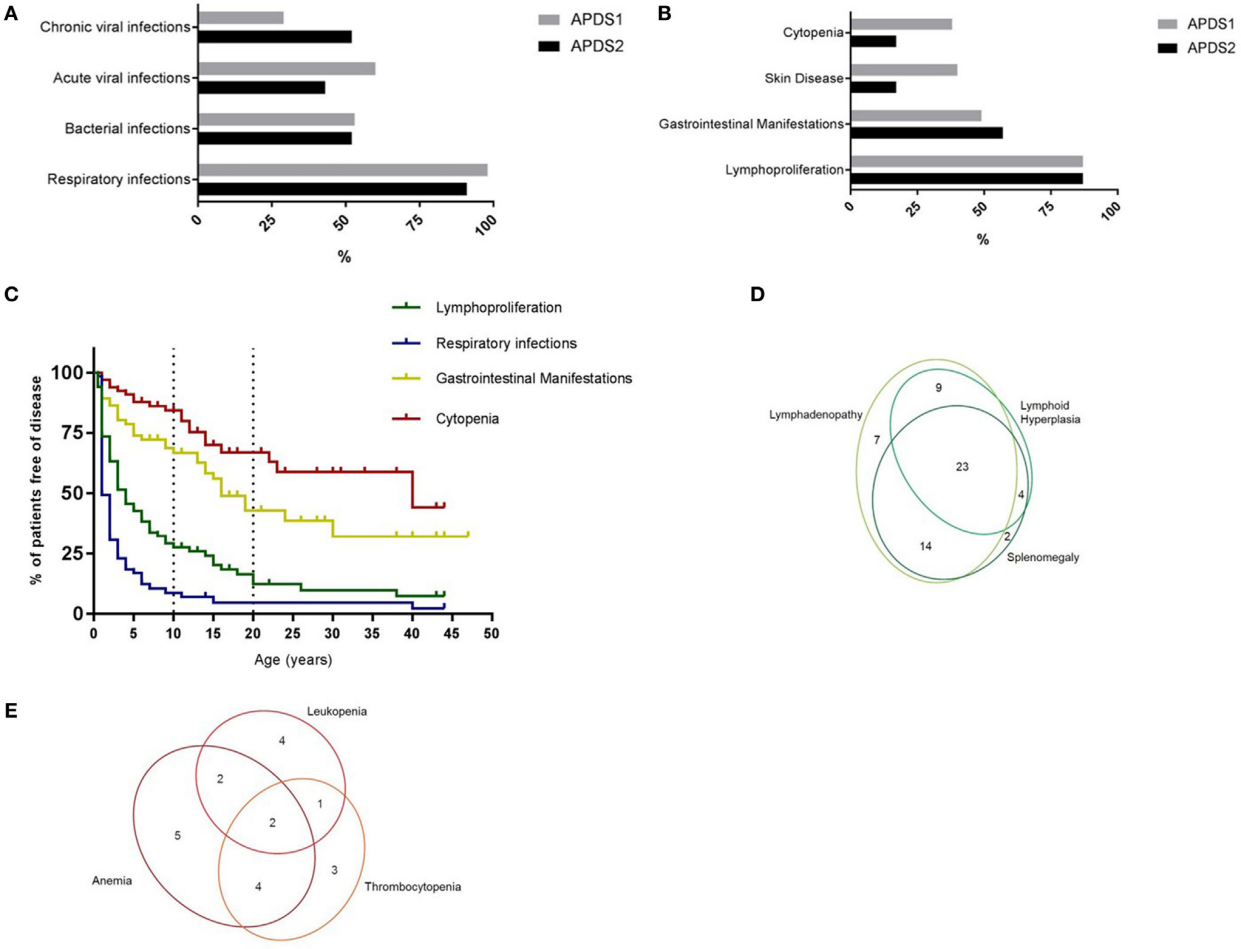
**(A)** Incidence of infections in APDS1 and APDS2 patients. **(B)** Incidence of manifestations of immune dysregulation in APDS1 and APDS2 patients. **(C)** Evolution of disease manifestations over time. Information regarding age at onset available for: respiratory infections *n* = 62/65, lymphoproliferation *n* = 59/59, gastrointestinal manifestations *n* = 33/35, cytopenia *n* = 20/21 patients. **(D)** Diagram showing the different types of benign lymphoproliferative manifestations. **(E)** Diagram showing the different blood lineages affected in patients with cytopenias.

By December 2017, 77 patients had been enrolled in the APDS Registry, 51 with APDS1, and 26 with APDS2. Detailed clinical and immunological information of 68 patients [39 of them not published in the cohort papers (5, 6)] from 59 unrelated families was available for this initial analysis. Forty-five of these 68 patients were diagnosed with APDS1 (43 with the E1021K and 2 with the C416R mutation) and 23 with APDS2 (all with mutations leading to skipping of exon 11). At the time of evaluation, living patients (65) had a mean age of 17.9 years (range 3–47 years). The main clinical features reported in APDS1 and APDS2 are summarized in Figures 1A,B. As in the previously reported cohorts, recurrent respiratory infections were by far the most frequent manifestation, occurring in 96% of the patients. Upper respiratory tract infections, otitis media, and sinusitis were the leading diagnoses, and, importantly, 59% of the patients had experienced at least one episode of pneumonia. Cumulative retrospective data highlight that the respiratory infections begin very early in life, with almost all patients being affected by the age of 15 (Figure 1C). The registry data confirmed the previously described (5, 6) high incidence of bronchiectasis (28 patients out of the 55 who underwent a CT-scan), which was documented early in life (age range: 2–39 years; mean: 11.2 years). As already suggested by a previous retrospective review of the literature (7), the majority of patients with bronchiectasis had APDS1 (24 patients out of the 40 who had a CT-scan). Abnormal lung function was noted in 17 out of 35 patients who performed these tests. Acute viral infections (with varicella and herpes simplex) as well as chronic viral infections/reactivations were frequently documented in APDS1 and APDS2 patients (Figure 1A). The most frequently reported chronic infection in both cohorts was Epstein–Barr virus infection (16/68). Among the non-respiratory bacterial infections, the most frequent was infectious lymphadenitis (14/68). Five patients suffered from chronic mucocutaneous candidiasis and three developed local infection following vaccination with bacillus Calmette–Guérin. Consistent with the two published cohorts, chronic non-neoplastic lymphoproliferation was reported in the majority of patients (87%). Persistent peripheral lymphoproliferation, splenomegaly, and lymphoid hyperplasia were frequent and they were often concomitantly reported in the same patients (Figure 1D). Across the cohort, lymphoproliferation occurred with later onset than respiratory infections (Figure 1C) but preceded gastrointestinal manifestations and the development of autoimmunity.

Benign lymphoproliferation may be difficult to distinguish from malignant disease, the risk of which is increased in APDS patients. Eight of the registry-documented patients (5 APDS1, 3 APDS2) developed lymphoma between the age of 11 and 25 years, including two patients with Hodgkin lymphoma, one of whom subsequently developed an intestinal diffuse large B-cell lymphoma. Six patients were diagnosed with non-Hodgkin lymphomas (two diffuse large B-cell lymphomas, one anaplastic lymphoma, one marginal zone lymphoma, two without detailed histologic information). Five patients achieved a complete remission on treatment, one patient achieved only a partial remission, one patient was still under treatment at the time of registration, while in the remaining case, the lymphoma was sadly fatal. One of these eight patients also had a benign ovarian serous cystadenoma. One patient developed a B-cell chronic lymphocytic leukemia at the age of 40 years. In addition to the established high incidence of hematological malignancy, 2 cases of solid organ malignancy or pre-malignancy were noted: one case of ductal breast carcinoma-*in situ* (diagnosed in an APDS2 patient at the age of 33) and one case of rhabdomyosarcoma (diagnosed in an APDS1 patient at the age of 13).

Gastrointestinal manifestations were the third most frequent disease manifestation (51%) and across the cohort occurred before the other features of immune dysregulation, such as cytopenias or arthritis, but typically much later than the respiratory infections and the benign lymphoproliferation (Figures 1B,C). Small or large bowel inflammation was histologically confirmed in 17 patients, in 11 of them by the age of 10 years. Granulomas were reported in only one patient. Protracted diarrhea with no identified underlying cause was the second commonest reported gastrointestinal problem and was often severe enough to require hospitalization. Two patients were diagnosed with autoimmune hepatitis but no cases of sclerosing cholangitis were reported, in contrast with the two patients reported by Coulter et al. (5) and the two reported by Hartman et al. (8). Of note, 14/68 patients of the APDS-Registry cohort had eczema. Elkaim et al. (6) noted only three APDS2 patients with chronic eczema and no inflammatory skin disease was mentioned in the published APDS1 cohort (5). Cytopenias were the fourth major disease manifestation affecting around 30% of patients, usually later in life (Figures 1B,C) than the other main features and frequently affecting multiple blood lines (Figure 1E). The autoimmune origin of the cytopenias could be documented in the majority of the patients. Other autoimmune diseases were also reported, all occurring after the age of 10 years: two patients had autoimmune thyroiditis, three had arthritis, and three glomerulonephritis.

Concerning non-immunological manifestations, short stature (>2 SD) was reported in 11 patients, with a predominance of APDS2 individuals (8/13), consistent with previous reports (6, 7). Neurodevelopmental delay was diagnosed in three patients. Specific neuropsychiatric disorders were also reported: one patient had Asperger Syndrome, one had autism, one suffered from a mixed anxiety and depression disorder, and two other patients had mild disorders of speech and language development. It is unclear if these findings reflect the impact of a severe physical illness or the impact of enhanced PI3Kδ signaling in the central nervous system.

### Immunological Abnormalities

One of the objectives of the ESID-APDS registry is to collect immunological data prospectively. An initial analysis of the immunological profile in the registry cohort confirmed the already published T- and B-cell alterations. No clear difference between APDS1 and 2 was detected in the current cross-sectional data set. In the future, the longitudinal collection and analysis of these data will offer the possibility to explore associations between specific disease manifestations and immunological alterations, to evaluate the response of immunological alterations to the different types of treatment, and to establish the predictive value of immunological parameters for disease prognosis.

### Current Therapies

**Figure 2:**
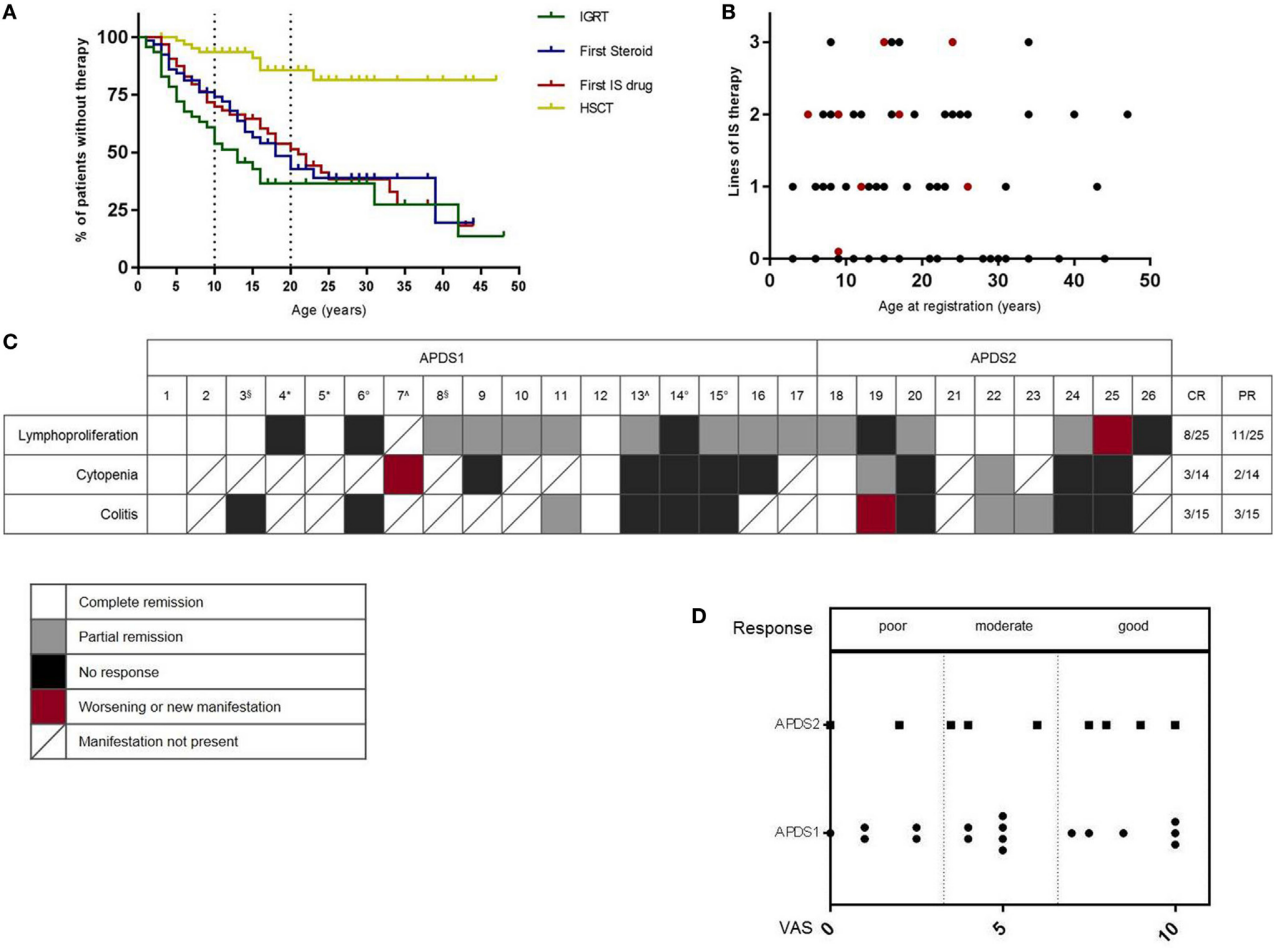
**(A)** Use of treatment modalities over time. IGRT, immunoglobulin-replacement-treatment; IS, immunosuppressive drug; HSCT, hematopoietic stem cell transplantation. Information regarding age at first therapy available for: IGRT *n* = 28/44, steroid therapy *n* = 31/31, IS therapy *n* = 35/36, HSCT = 8/8. **(B)** Number of lines of immunosuppressive treatments (steroids, immunosuppressive drugs, rituximab) by the time of registration; red: patients who had undergone HSCT by the time of registration. **(C)** Response to rapamycin treatment. *White*: complete response; *gray*: partial response; *black*: no response; *red*: worsened or new manifestation; *boxes with a diagonal*: manifestation not present in this patient. CR, complete remission; PR, partial remission. Rapamycin stopped because of: *non-compliance, °inefficiency, ^^^side effects, ^§^clinical trial. **(D)** Overall clinical benefit (Visual Analog Scale) according to physician’s evaluation.

Supportive therapy is a key component of the management of APDS patients. In the APDS-registry, 54 patients received antibiotic prophylaxis, whereas only eight received antifungal prophylaxis, which appears justified given the absence of reported invasive fungal infections. IGRT was administered in 44 patients (28/45 APDS1, 16/23 APDS2), was in general very well tolerated, and was started early in life (Figure 2A), mirroring the early presentation with respiratory infections. The majority of patients also received immunosuppressive treatments. Thirty-one patients received corticosteroids and 27 of them showed at least a partial clinical benefit. More than half had received steroid treatment by the age of 20 (Figure 2A). Thirty-six patients received other immunosuppressive drugs, including azathioprine (*n* = 1), mycophenolate (*n* = 3), cyclosporine (*n* = 5), or rapamycin (*n* = 27); clinical benefit was reported in 28 of these patients. Rituximab was given to eight patients, with clinical benefit in all. Figure 2B illustrates the multiple lines of immunosuppressive treatments (steroids, immunosuppressive drugs, or rituximab), which had already been received by patients by the time of enrollment into the registry. Five patients underwent splenectomy (4 APDS1 and 1 APDS2) because of cytopenias or splenomegaly and 25 patients (12 APDS1 and 13 APDS2) underwent tonsillectomy (age range: 1–12 years), with clear benefit in only seven of them. The only available curative option is hematopoietic stem cell transplantation (HSCT) and the first experiences in this field have been published (9). Among the patients in the registry, 8/68 patients had undergone HSCT (7 APDS1 and 1 APDS2) by the time of registration (Figure 2A), with fatal outcome in one.

### Rapamycin Therapy in APDS

Consistent with activation of mTOR signaling downstream of the activated PI3Kδ, patients with APDS may benefit from rapamycin (2). In the APDS2 cohort-paper (6), six patients had been treated with rapamycin, but the time of follow-up was too short to evaluate the response to treatment in four of them. Six of the patients in the reported APDS1 cohort (5) were treated with rapamycin for benign lymphoproliferation; five of them had a treatment response, but in one case, the therapy was stopped due to side effects. Additional case reports of rapamycin therapy have also been published (7, 10). In the ESID-APDS-registry cohort, rapamycin was the most frequently used immunosuppressive drug. We, therefore, decided to evaluate the experience with rapamycin (Sirolimus) in 26 patients (1 patient was not included because treatment was started and terminated before the diagnosis of APDS and the response to therapy was not well documented), 17 with APDS1, and 9 with APDS2. The main indications for treatment were lymphoproliferation, colitis, and/or cytopenia. Physicians were asked to judge the degree of severity of each manifestation as mild, moderate, or severe at the start of therapy, following 3–6 months of treatment and at the latest follow-up (average time of therapy monitoring: 1.6 years). Overall response judged by the physician visual analog scale was good in 10, moderate in 9, and poor in 7 (Figure 2D). Lymphoproliferation showed the best response (8 complete, 11 partial, 6 no remission), while bowel inflammation (3 complete, 3 partial, and 9 no remission) and cytopenia (3 complete, 2 partial, 9 no remission) responded less well, as shown in Figure 2C. Notably, of the eight patients who were on steroids at initiation of treatment with rapamycin (No. 1, 7, 9, 13, 19, 22, 23, 25), seven were able to stop steroids and one (No. 25) was able to reduce the dose. Two patients (No. 4, 5) stopped therapy because of poor compliance, in three cases (No. 6, 14, 15), the reason for cessation was lack of efficacy. Two patients (No. 7, 13) suffered from side effects (severe headaches, anorexia, renal toxicity) that led to the complete interruption of the treatment, whereas in three cases, the therapy was paused because of side effects (aphthous ulcers, liver toxicity, renal toxicity) but could be started again. Two patients (No. 3, 8) stopped despite efficacy because of enrollment in a clinical trial with PI3Kδ inhibitors. In two other individuals (No. 11, 12), treatment was interrupted after prolonged usage; in one patient (No. 20), this was due to the patient planning for pregnancy and, in another (No. 19), it followed the development of a lymphoma. Of note, three patients (No. 14, 18, 25) received also Rituximab during and one (No. 10) shortly before the treatment with rapamycin. One patient (No. 20) concomitantly received Adalimumab because of arthritis. Interestingly, some patients did not show any relevant alterations in the disease manifestations after 3–6 months of therapy but did show either improvement (No. 1, 8, 10, 18, 22, 23) or worsening (No. 6, 14, 19) after a longer period of observation on treatment (about 2 years).

## Discussion

We present an initial analysis of the prospective ESID-APDS registry, a longitudinal cohort study of patients with APDS1 and APDS2. This overview expands the known information regarding the clinical manifestations of the disease by adding the aspect of the evolution of the features over time. The emerging picture is the one of a PID characterized by the early occurrence of respiratory infections (mostly upper respiratory infections), followed by the development of chronic benign lymphoproliferation and subsequently other features of immune dysregulation, in particular, gastrointestinal manifestations and autoimmune cytopenias. We again noted the higher incidence of bronchiectasis in APDS1 compared with APDS2 patients; however, the numbers remain small and differences in CT uptake cannot be excluded as a confounder. However, this observation may stimulate future studies of the roles of the *PIK3CD* and *PIK3R1* genes and their proteins in the respiratory system. In the future, further analysis of the clinical evolution in this prospective cohort will allow better definition of long-term prognosis for this disease. In addition, the correlation of clinical features with the immunological abnormalities and their relationship with outcome parameters will help defining clinical and biological biomarkers of outcome.

The choice of treatment is a key issue in these patients who often present with severe concomitant manifestations not only of immunodeficiency but also of immune dysregulation. According to the registry, the combination of supportive therapy to prevent recurrent infections and the immunosuppressive treatment of immune dysregulation is often initiated early in life, with many patients undergoing multiple treatments. Rapamycin inhibits the biologically relevant downstream PI3K effector mTOR pathway, and it has been widely used with good efficacy in other PIDs, in particular, autoimmune lymphoproliferative syndrome (11, 12). Our interrogation of the ESID-APDS registry aligns with previous reports (7, 10) in suggesting that rapamycin reduces the severity of benign lymphoproliferative disease also in APDS. However, a less satisfactory response was documented regarding the non-lymphoproliferative manifestations, in particular, intestinal disease and cytopenias, which can be highly detrimental for the patients’ quality of life. It is important to relate these registry results to the first results of targeted therapy with the PI3Kδ inhibitor leniolisib that have recently been published (13). In the first six patients, the drug showed an excellent control of the lymphoproliferation (6/6 patients) and in part also improved the cytopenias at the end of treatment (day 84). Three of the six patients normalized their thrombocytopenia, one patient resolved his anemia, and three of four patients improved their lymphopenia, while there was no correction of the neutropenia observed in two patients; however, respiratory and gastrointestinal symptoms and outcomes were not reported in this study. Furthermore, our registry analysis highlighted that also colitis and skin disease can cause significant symptoms in these patients and should, therefore, be carefully evaluated in future clinical studies on novel therapies, particularly given previous reports of colitis associated with PI3K inhibitors (14). Longitudinal data capture on APDS patients in the ESID-APDS registry will be critical to observe the long-term benefits and/or side effects of these therapies, in particular, their effect on the incidence of lymphomas. It is noteworthy that one patient developed lymphoma while taking rapamycin. Another key question, where the registry will be helpful, is the question if and when to perform HSCT. The analysis of this question will profit from the principles established in the P-CID study, a prospective natural history study on profound combined immunodeficiency in which matched pairs of transplanted and non-transplanted patients with similar disease burden and immunological alterations are followed (15).

Finally, an attractive goal for the registry is to involve patients and their families directly in data acquisition. This could in the future allow collecting information about the quality-of-life of APDS patients, thus ameliorating the evaluation of the disease burden in all its complexity. In summary, thanks to the collaborative work of the participating centers, the ESID-APDS registry will comprise a valuable resource for physicians dealing with this disease and for shaping future research questions.

## Ethics Statement

The study is carried out in accordance with the recommendations of Section 15 of the Code of Conduct of the General Medical Council of Baden-Württemberg, Germany. The protocol was approved by the Ethics committee of the University of Freiburg (IRB approval No. ESID registry: 493/14; IRB approval No. APDS registry: 458/15). All subjects gave written informed consent in accordance with the Declaration of Helsinki.

## Author Contributions

MM collected analyzed and interpreted data and wrote the manuscript. HA, AA, ALA, OA, CB, SAB, FB, HB, MB, SOB, CC, ANDC, PC, MC, ANIC, FC, TC, LD, JE, SF, AF, MG, LH, MH, SJ, EK, AK, DK, BG, HL, NM, TM, FM, DM, AM, ON, BN, PO, AO, JP, CP, SP, JR, SS, ALS, ANS, SS, ASH, MS, PS, AUS, FS, WR, FT, JM, KW, AW, and PW repeatedly referred and registered patients. AN, GK and AU coordinated the registry. SR and RS provided the export data from the online-registry and gave informatic support. SK, ALC, and SE interpreted the data and wrote the manuscript. All the authors edited the manuscript.

## Conflict of Interest Statement

The APDS registry is supported by the pharmaceutical companies Novartis, GlaxoSmithKline, and UCB UK, who have financed development and maintenance of the online level 3 documentation section for APDS as well as project management including ethics submission in all participating countries, data management, and quality controls. The financial support also allows some reimbursement of documentation activities for the participating centers. For those patients who have specifically agreed to this in the registry consent, anonymized data from the APDS Registry are available to industry partners for their purposes (e.g., designing a drug trial or data submission for regulatory approvals).
